# Supplementary figures and images for: Dual contribution of ASIC1a channels in the spinal processing of pain information by deep projection neurons revealed by computational modeling
Source: PLoS Comput Biol. 2023 Apr 17;19(4):e1010993. doi: 10.1371/journal.pcbi.1010993 (PMC10109503; doi:10.1371/journal.pcbi.1010993)

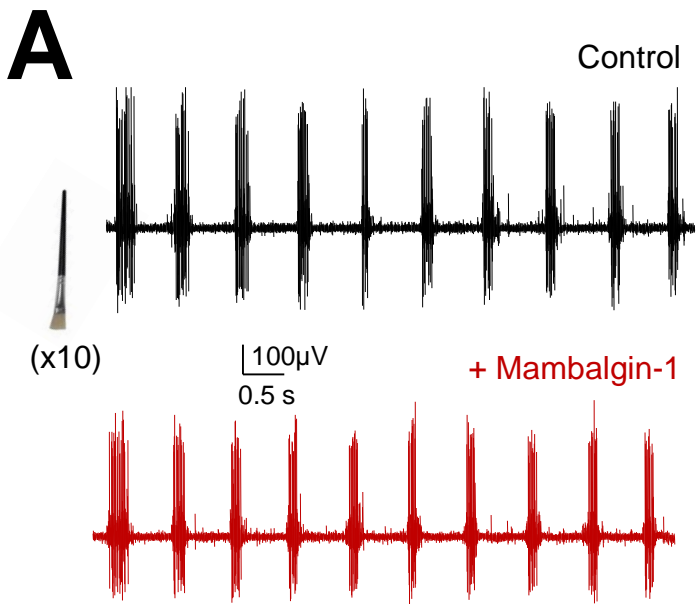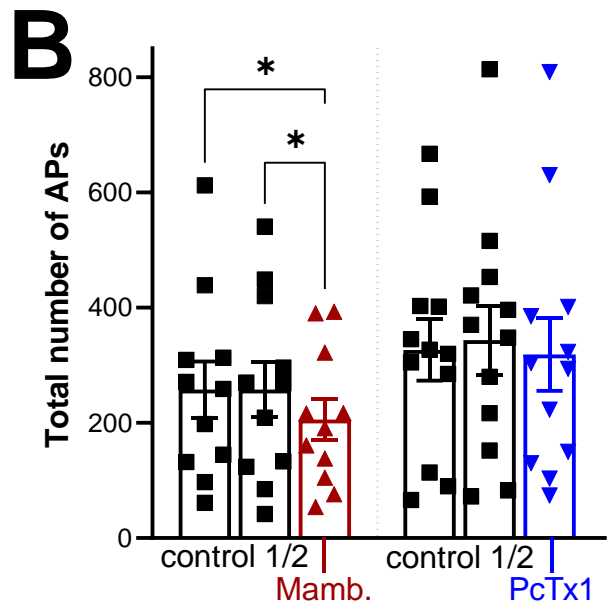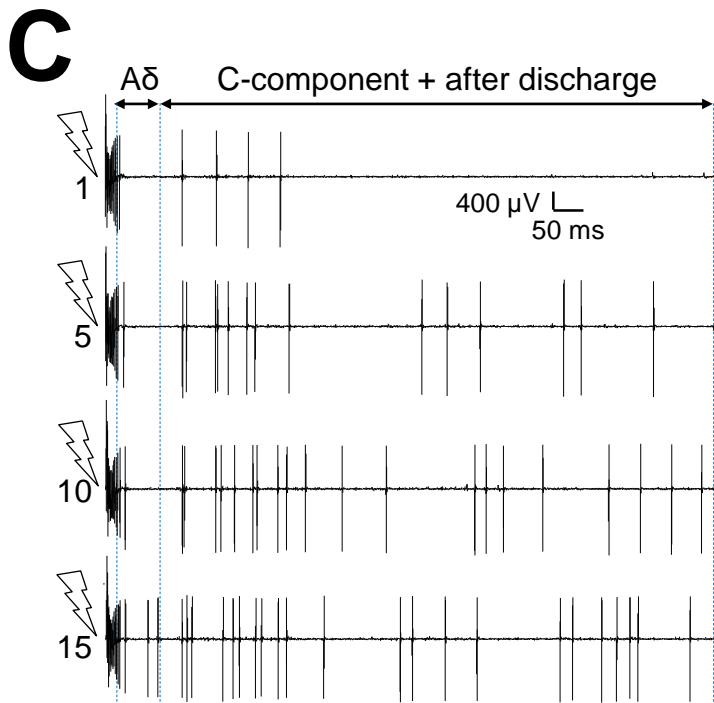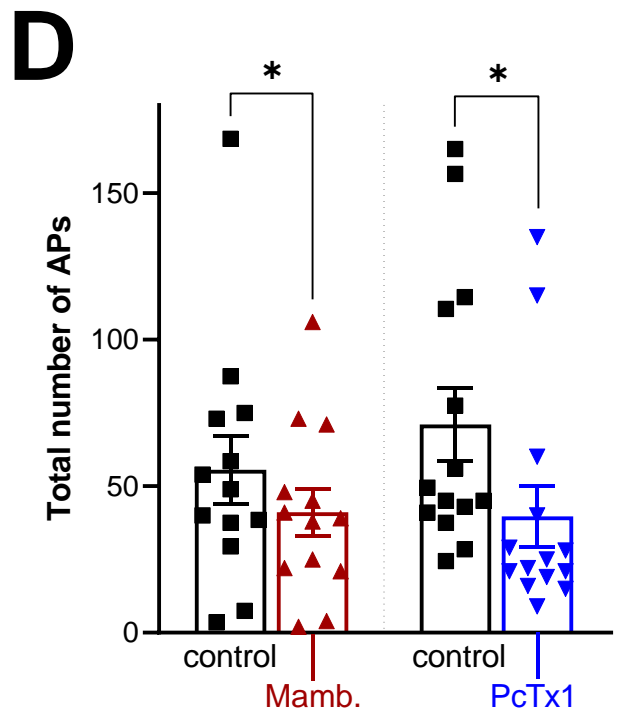

SUPPLEMENTARY FIGURE 1

Supplement: S1 Fig — A, Typical recordings obtained following stimulation of a WDR neuron receptive field by 10 repetitive brushings (non-noxious stimulations), before (control) and after a 10-min application of mambalgin-1 (30μM) at the spinal cord level. B, The total number of AP evoked during the brushing protocol described in A are compared before (control 1 and 2, which represent two brushing experiments that were performed consecutively at 10 min intervals) and after applications of either mambalgin-1 or PcTx1 30μM (n = 11–12, p<0.05, one-way ANOVA tests followed by Dunnet’s mutiple comparison tests). C, Typical recordings showing the activity of a WDR neuron during a windup protocol (16 repetitive electrical stimulations at 1Hz). Only the recordings obtained at stimulation 1, 5, 10 and 15 are represented. The vertical dashed lines represent the time ranges where the activity of the WDR is considered to be evoked by Aδ or C fibres. D, Total number of AP evoked by Aδ during windup protocols before (control) after applications of either mambalgin-1 or PcTx1 (n = 13–14, *, p<0.01, paired t test). (PDF) [file pcbi.1010993.s001.pdf]

**A**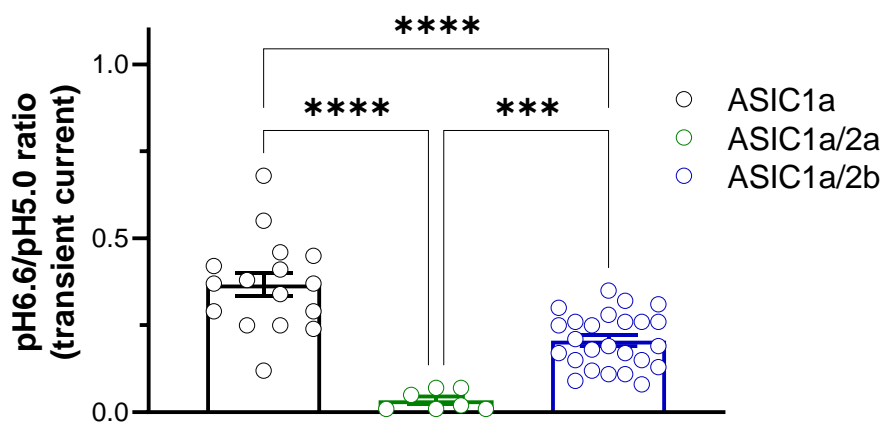**B**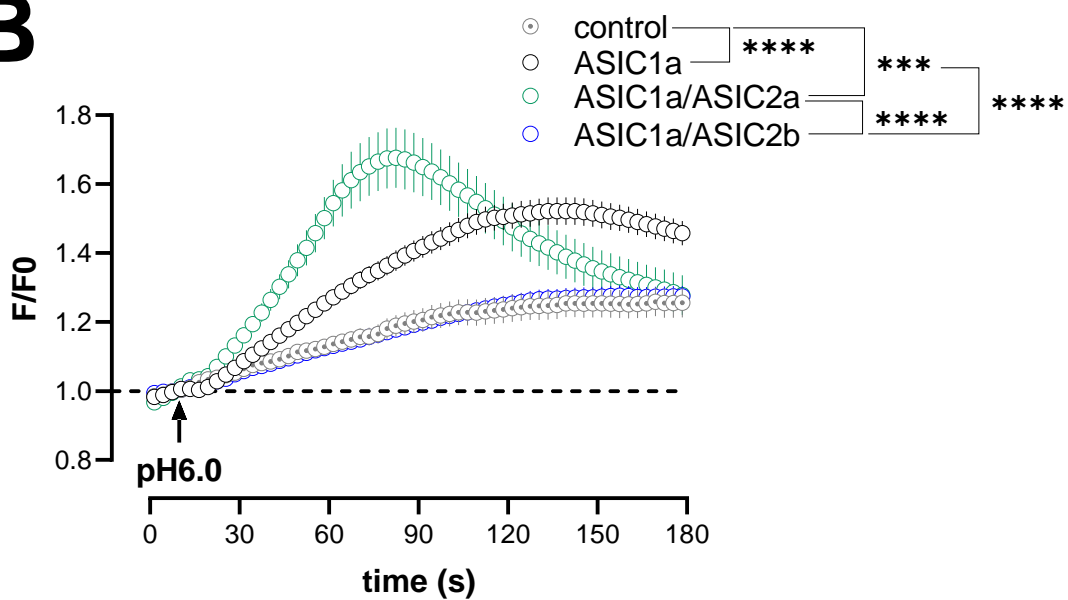**SUPPLEMENTARY FIGURE 2**

Supplement: S2 Fig — A, Peak current ratios (pH6.6/pH5.0) obtained from whole-cell patch-clamp experiments performed in HEK293 cells transfected with either ASIC1a alone, ASIC1a+ASIC2a or ASIC1a+ASIC2b (n = 7–25, One-way ANOVA followed by a Tukey’s post hoc test with ****p<0.0001). B, Fluorescence ratio (F/F0) measured in HEK293 transfected cells loaded with the fluo4 calcium probe following a 5-seconds acidification of the extracellular medium from pH7.4 to pH6.0. The arrow indicates the time at which the pH6.0 acidification was applied (n = 88, 125, 35 and 175 for control, ASIC1a, ASIC1a/ASIC2a and ASIC1a/ASIC2b respectively, Two-way ANOVA followed by a Tukey’s post hoc test with ***p<0.001 and ****p<0.0001). (PDF) [file pcbi.1010993.s002.pdf]

**A**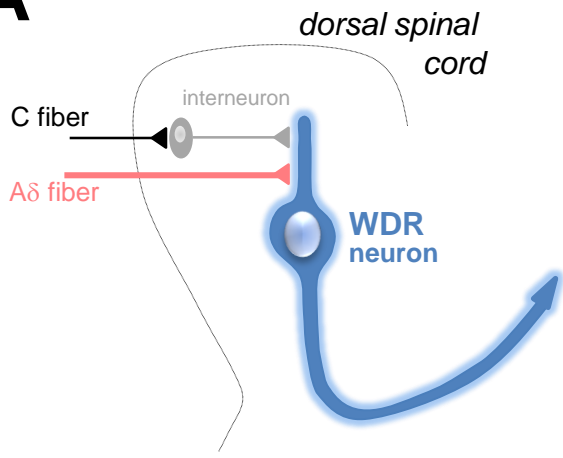**B**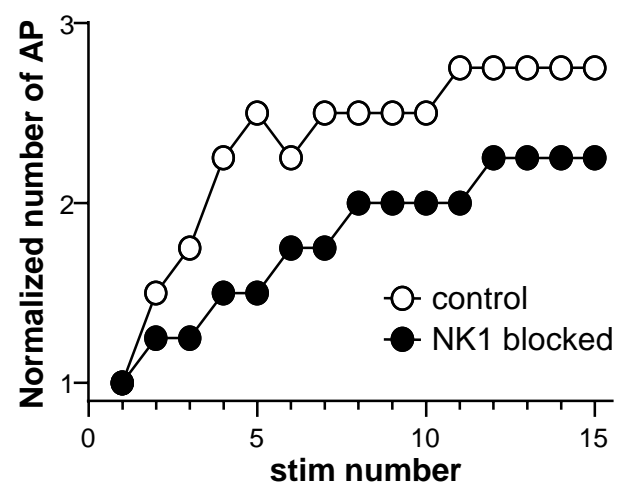**C**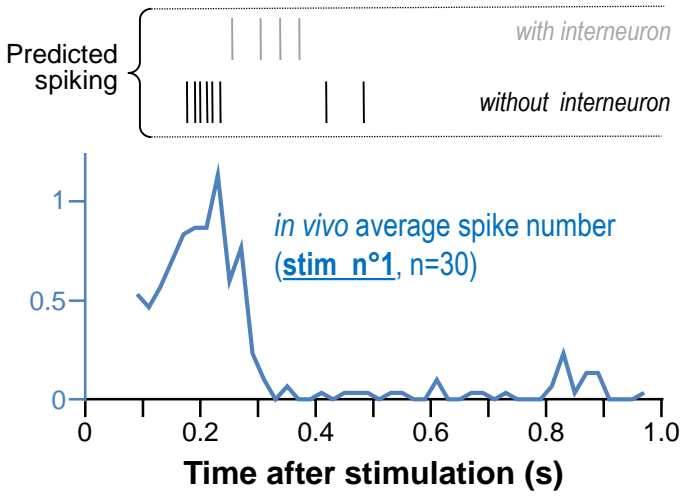**D**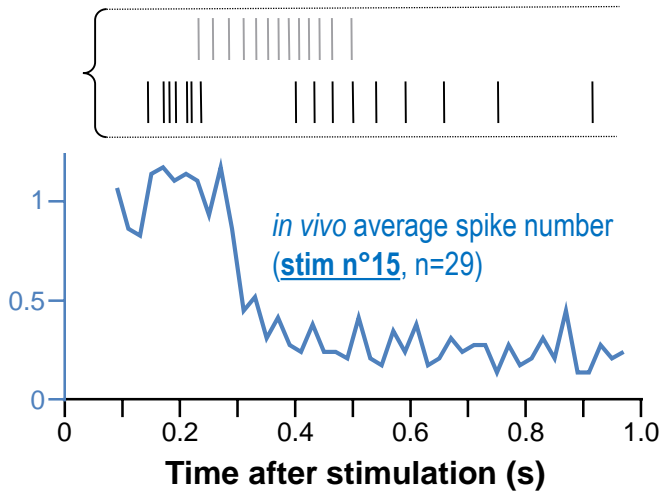

**SUPPLEMENTARY FIGURE 3**

Supplement: S3 Fig — A, Initial model developed by Aguiar and colleagues [41]. B, Using Aguiar’s model allows us to reproduce the results on windup when NK1 receptor parameters are removed. C-D, Spiking time profiles obtained for the first (stim1) and the fifteenth (stim 15) of the windup protocol (repetitive stimulations at 1Hz). Experimental data (blues curves), representing the mean number of AP per 20ms as a function of time (data from 29–30 neurons), are compared to data predicted by the Aguiar model, with (upper gray marks) or without (upper black marks) the interneuron between C-fiber and WDR neuron. (PDF) [file pcbi.1010993.s003.pdf]

**A**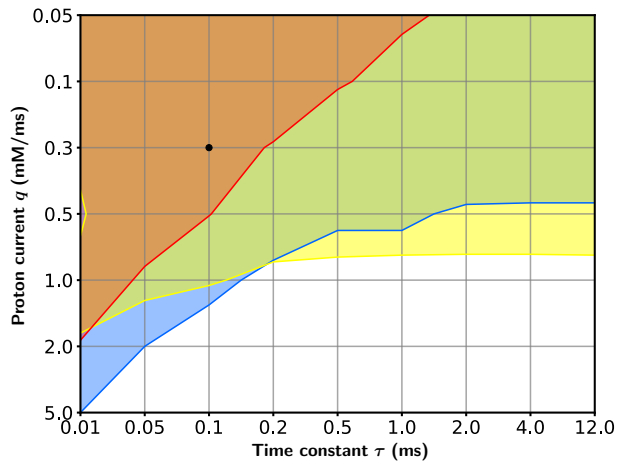**B**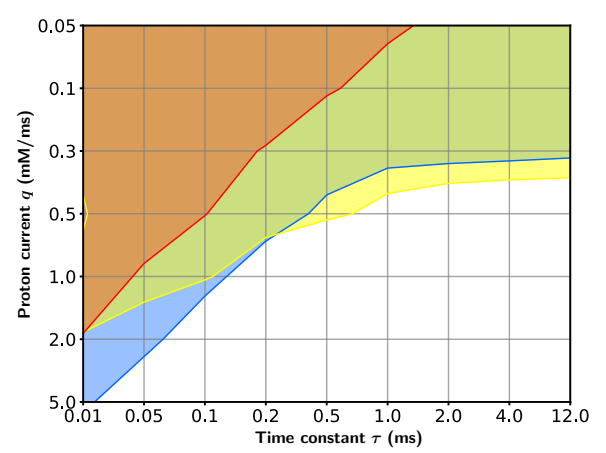**C**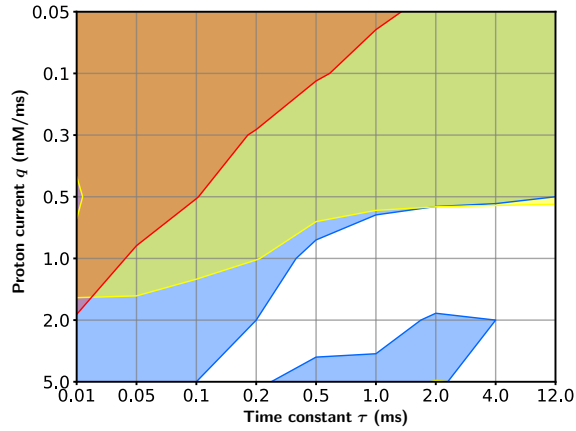**D**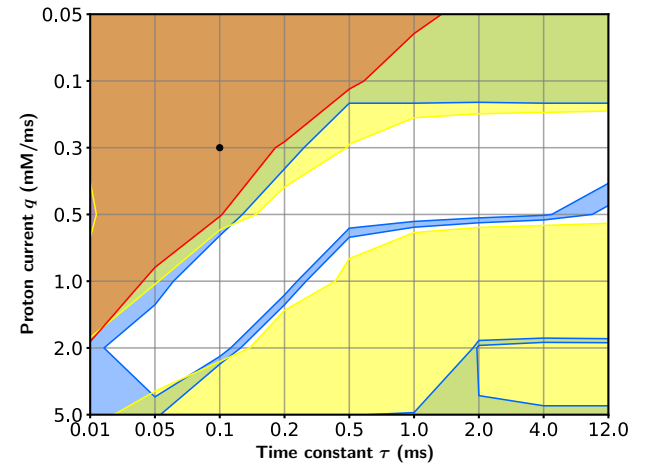**E**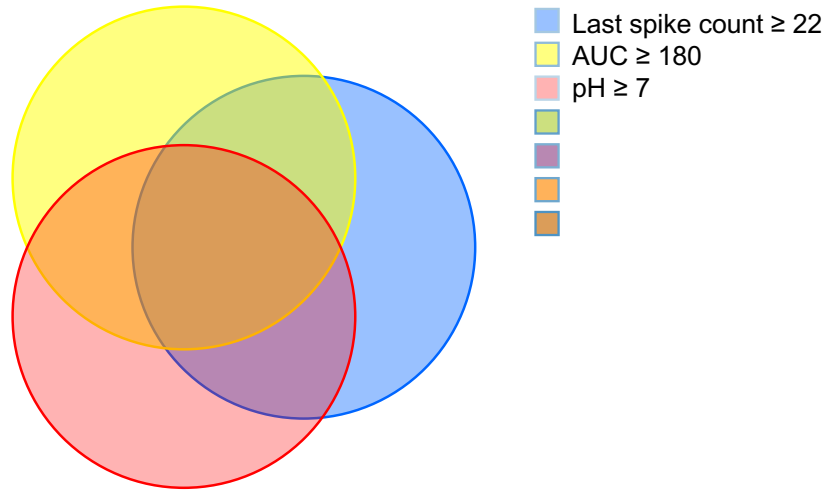**SUPPLEMENTARY FIGURE 4**

Supplement: S4 Fig — Grid searches for the values of q and τ, the two least constrained parameters of the synaptic cleft pH model, for different models of τh. The time constant τ has no direct physiological meaning and is unconstrained. The proton current q is considered acceptable below the maximum 2mM/ms, which represents the effect of an outward current of 250pA/pF (as was observed in HV1-transfected HEK293 cells depolarized to +90mV in [56)) through a membrane of capacitance 2,4μF/cm2 as measured for spinal cord neurons by [57], into a synaptic cleft of 20nm width. We represent, on a q vs. τ plane, the zones in which (i) pH remains within a physiologically plausible range (ε7, red zone), (ii) windup matches experimental data (ε 180, yellow zone) and (iii) the number of spikes elicited by the last stimulation matches experimental data (ε22, blue zone). Conditions (ii) and (iii) together constrain the shape of the windup curve. Black dot represents our chosen model. The maximal ASIC conductance is g = 0.2nS for A, B, C. The colored lines mark the border of each zone, and the colors resulting from the overlap of several zones are shown in E. The red zone represents parameters sets for which the pH remains ε7 over 100 stimulations, in order to exclude parameter choices for which the pH only stays within the physiological range because the simulation is interrupted before pH drops further, rather than because the pH parameters are physiologically valid on the long run. Because the available experimental data for τh in the native homomeric model was insufficient to infer any functional form for τh as a function of pH, different functional forms were tested: (A), a Gaussian fit as described in the Methods to reproduce a hump-shaped τh as proposed for the heterologous model [42]; (B), an affine form τh = max (0, −a pH+b), with a = 160.4 and b = 1195.32. (C), a piecewise-affine form with a maximum at the same pH value as the m∞*h∞ curve, τh=max(0,a1pH−b1)ifpH≤7.37andτh=max(0,a2pH+b2)ifpH≥7.3 [file pcbi.1010993.s004.pdf]

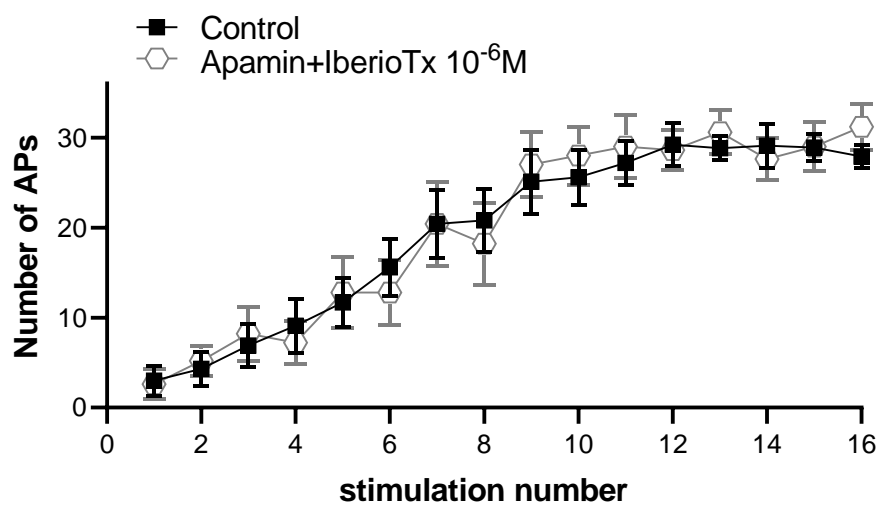

**SUPPLEMENTARY FIGURE 5**

Supplement: S5 Fig — Part of Fig 5D only showing the effects of Apamin + IberioTx (2nd) compared to control (1st). (PDF) [file pcbi.1010993.s005.pdf]

**A**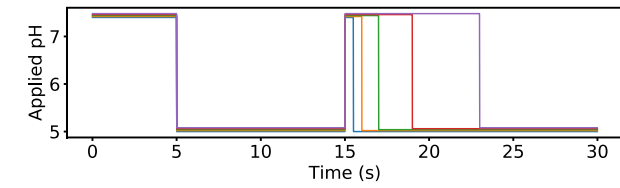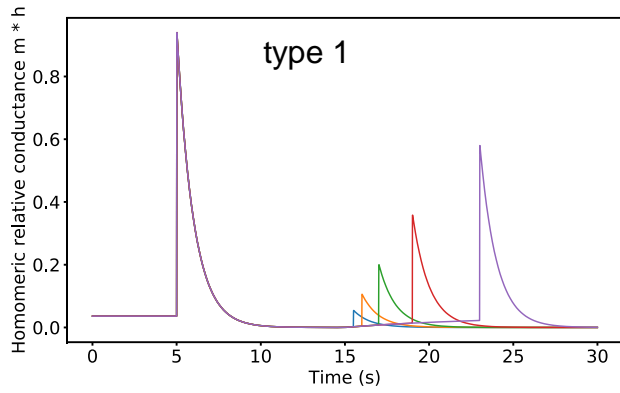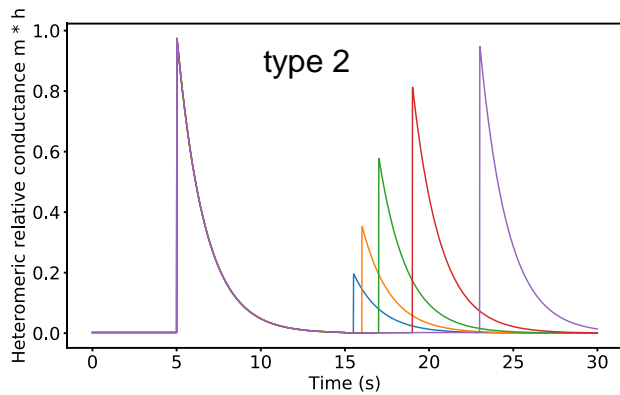**B**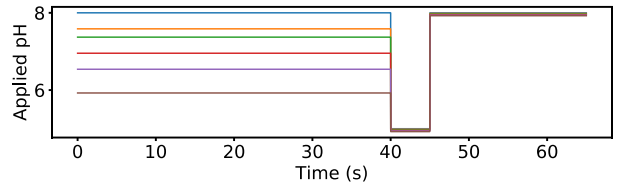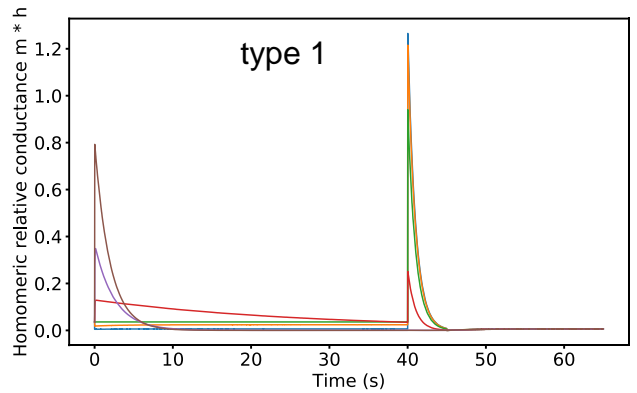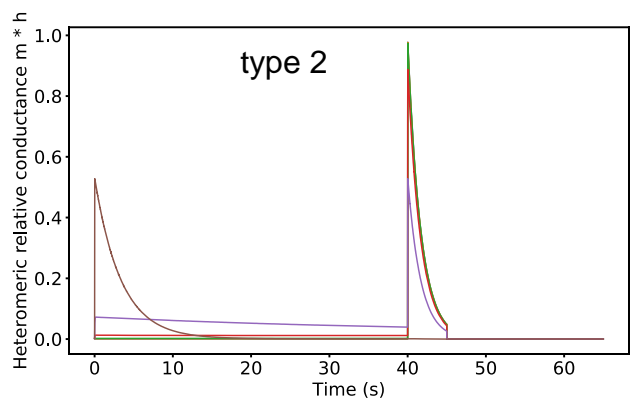**C**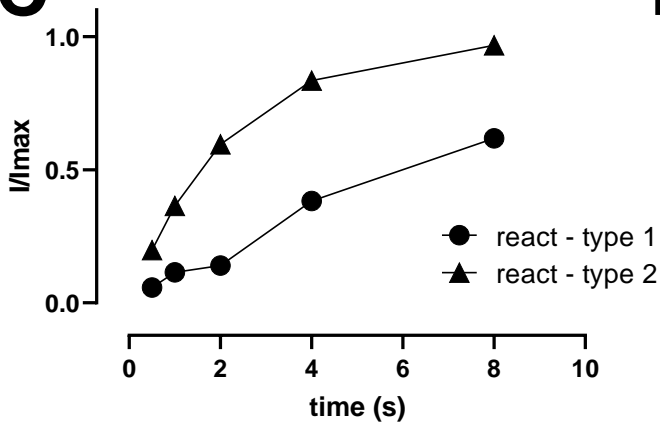**D**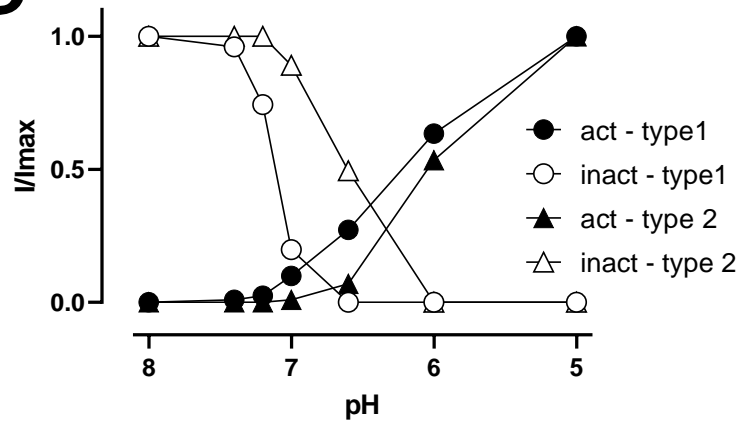**E**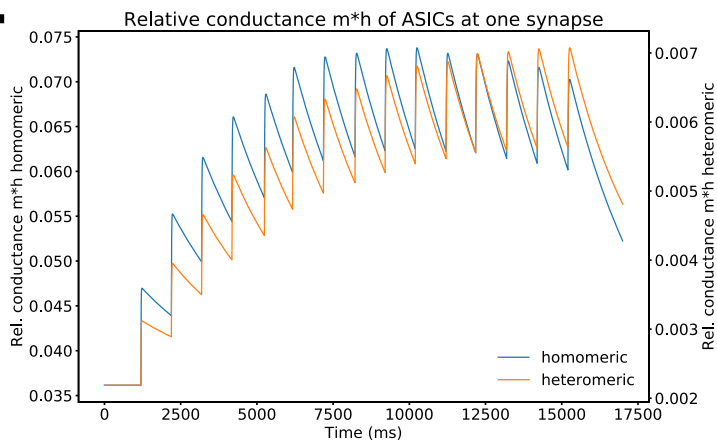**F**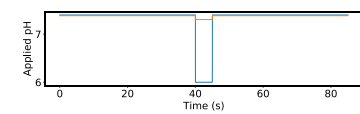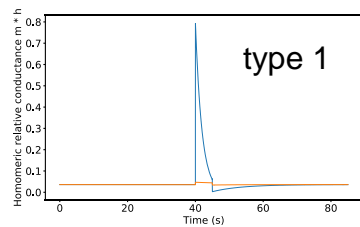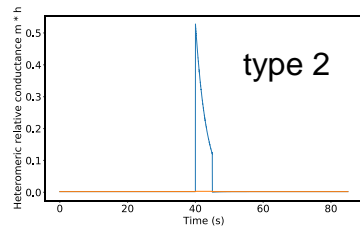**SUPPLEMENTARY FIGURE 6**

Supplement: S6 Fig — Simulation data representing the relative conductance obtained for the recovery from inactivation (A, C) and for the pH-dependent activation/inactivation (B, D) processes of native ASIC1a homomeric and ASIC1a/ASIC2 heteromeric models, which were elaborated with the type 1 and type 2 parameters, respectively. E, Relative conductance of ASIC channels of the two models in response to the synaptic cleft acidification modeled at the synapse (Fig 4B) during the simulation. Notice the different scales associated to the homomeric and heteromeric models, illustrating a difference of the conductance ranges over which the same qualitative behavior is observed. F, Relative ASIC conductance of the homomeric and heteromeric models when the channels are activated by a classical pH drop from pH7.4 to pH6.0 (blue) or 7.32 (orange). (PDF) [file pcbi.1010993.s006.pdf]

**A**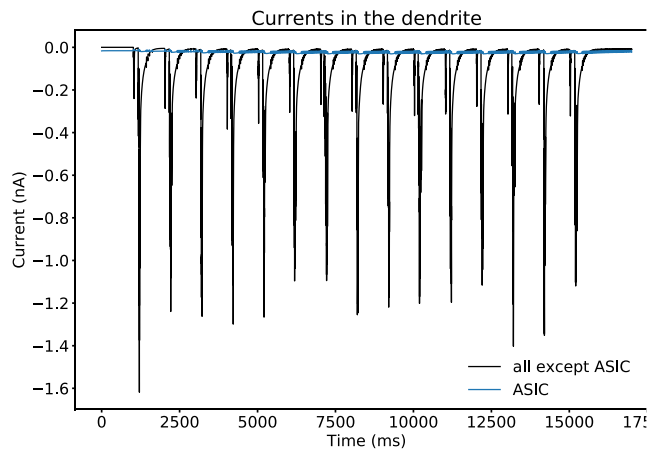**B**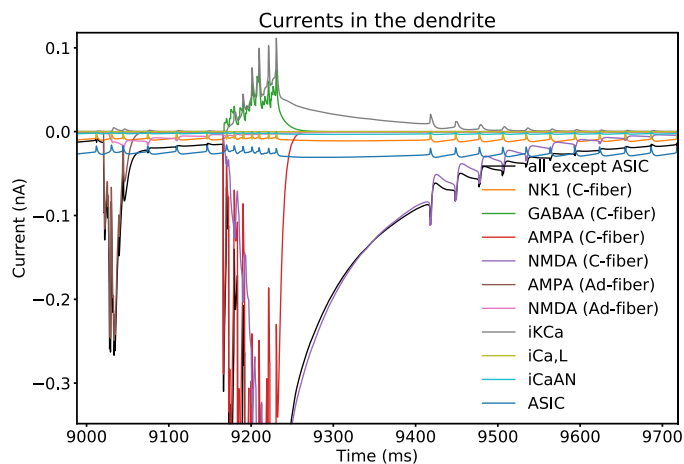**C**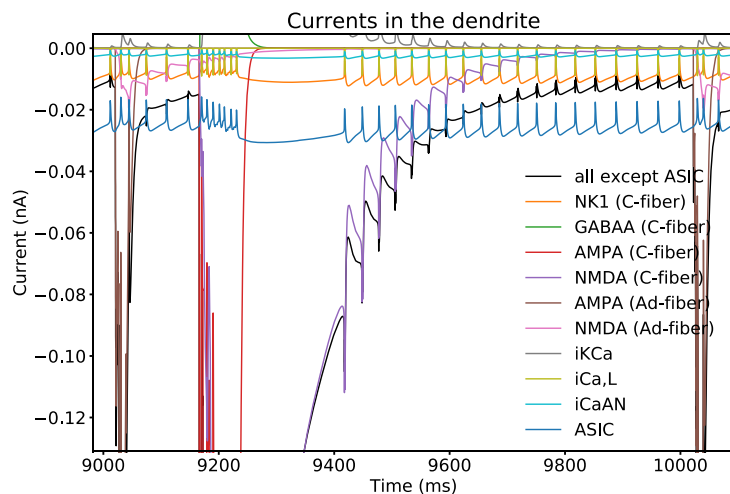**SUPPLEMENTARY FIGURE 8**

Supplement: S8 Fig — This figure shows the transmembrane currents in the dendrite of the WDR model in order to evaluate how ASICs compare with other currents playing a role in windup. The simulation uses the native homomeric model with maximal conductance 0.2nS. A, The ASIC current (blue) and the sum of all other transmembrane dendritic currents (black) over the whole duration of the simulation. The ASIC current is slowly activating and remains a small contribution compared to the overall current. B, C, Close-ups of the same graphs with added details of each current: blue curve is the ASIC current, black curve is the total dendritic current, which is the sum of all other shown currents. iKCa, calcium-activated K+ currents. iCa,L, L-type calcium currents. iCaAN, calcium-activated nonspecific cationic currents. The AMPA and NMDA currents, shown in brown, pink, purple and red, clearly dominate, as expected, the input to the dendrite. The ASIC current, shown in blue, is a sustained and increasing current. Its dynamic is rather similar to that of the NK1 receptor associated currents (orange) although the ASIC current amplitude is slightly bigger; this may explain that at low to moderate conductances, ASICs participate to windup as NK1s do (as long as the associated calcium influx is not too big). The ASIC currents also have a much bigger amplitude than the calcium-activated non-specific currents iCaAN (light blue). The dynamics of the L-type calcium channels (light green), only activating briefly at spike times, is quite different from that of ASICs, which might also explain why the calcium influx from ASICs may inhibit windup although L-type calcium channels participate to windup generation according to Aguiar et al. [41]. (PDF) [file pcbi.1010993.s008.pdf]

**A****alternative 1**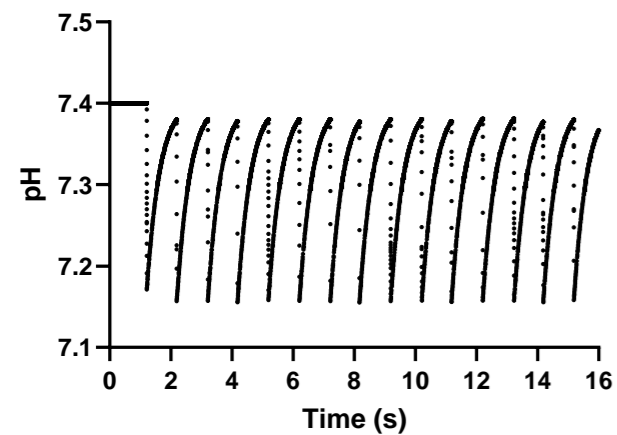**B**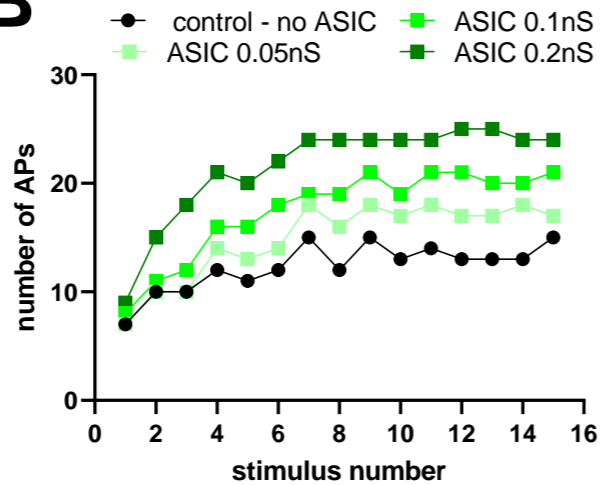**C**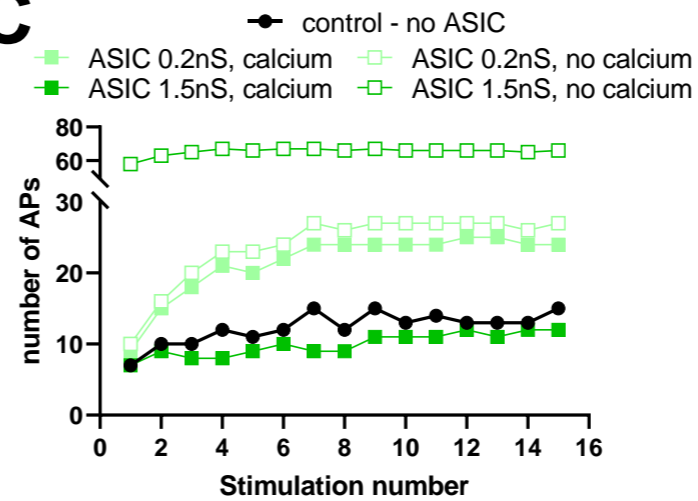**D**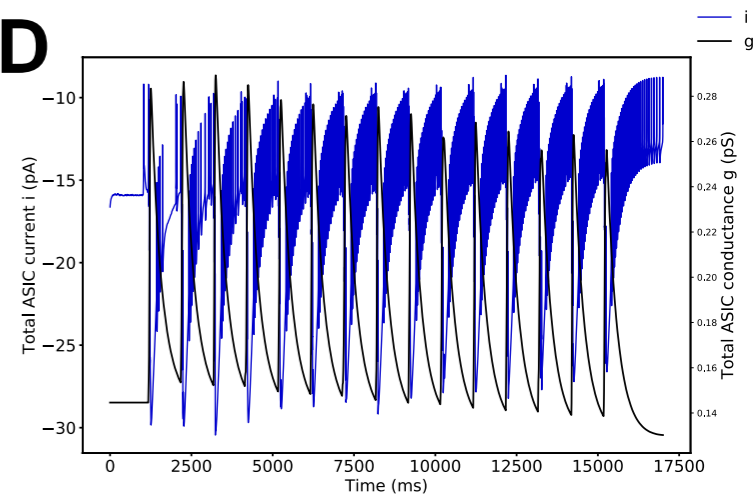**E****alternative 2**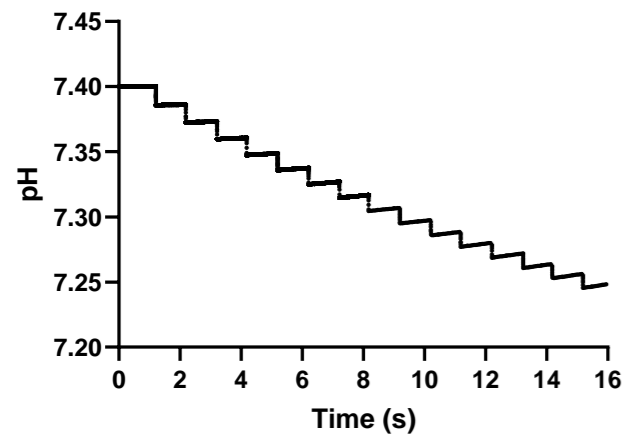**F**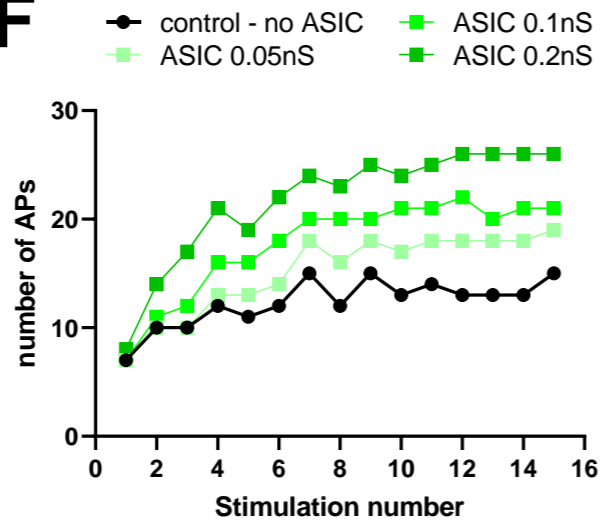**G**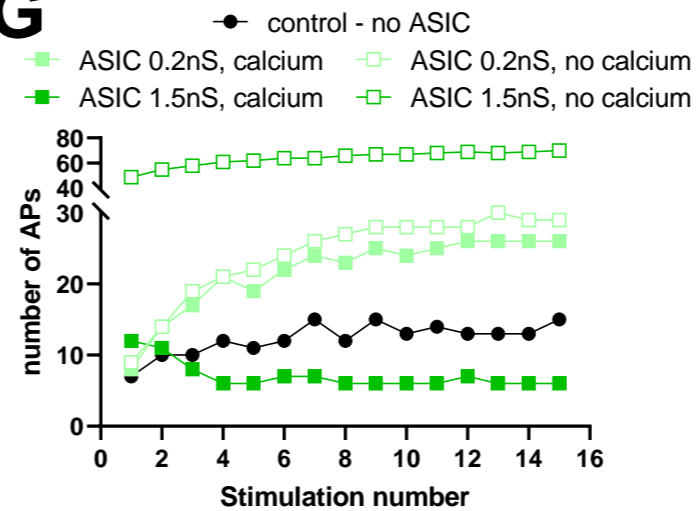**H**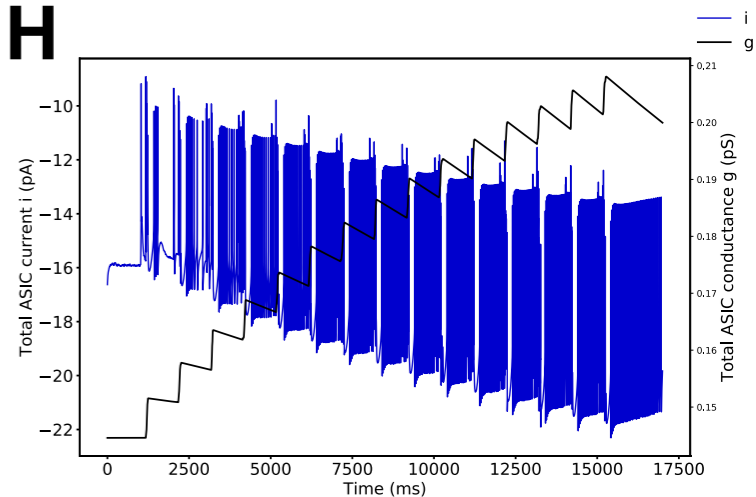**SUPPLEMENTARY FIGURE 9**

Supplement: S9 Fig — This figure reproduces the results of Fig 4B, 4C and 4D for other choices of the pH parameters q and yielding very different synaptic cleft pH dynamics. A, E, evolution of the synaptic cleft pH for two other sets of parameters (compared to Fig 4B) in the validity zone defined by S4 Fig: the alternative 1 parameter set is (q = 1mM/ms, τ = 0.01ms) which results in a very fast acidification time course; the alternative 2 parameter set is (q = 0.05mM/ms, τ = 1ms) which contrariwise results in a very slow acidification time course. B, F, reproduction of Fig 4C for alternative 1 and alternative 2 parameter sets respectively: progressively increasing the ASIC1a maximal conductance potentiates windup, or, from the experimental point of view, inhibiting ASIC1a channels reduces windup. C, G, reproduction of Fig 4D for alternative 1 and alternative 2 parameter sets respectively: higher ASIC maximal conductances inhibit windup, but only if ASICs are permeable to calcium. D, H, ASIC conductance and current during the windup protocol with alternative 1 and alternative 2 parameter sets respectively for maximal conductance 0.2nS. The time course of the ASIC currents depends on the set of parameters, but not the time course of the windup. Overall, these qualitatively similar results with drastically differing pH time courses show that the effect of ASIC1a channels on windup is brought on by their own intrinsic dynamics rather than by the dynamics of the synaptic cleft acidification. Synaptic cleft acidification is required to activate ASICs, but the resulting windup follows its own time scale, independent of the time course of the acidification. (PDF) [file pcbi.1010993.s009.pdf]
