# Supplementary material for: Dual contribution of ASIC1a channels in the spinal processing of pain information by deep projection neurons revealed by computational modeling
Source: PLoS Comput Biol. 2023 Apr 17;19(4):e1010993. doi: 10.1371/journal.pcbi.1010993 (PMC10109503; doi:10.1371/journal.pcbi.1010993)
Supplement: S7 Fig — In this figure, we represent the current amplitude of the iCaAN, iKCa and iCa,L currents in the WDR neuron dendrite during the simulation at medium (A, C; 0.2nS) and high (B, D; 1.4nS) conductances corresponding respectively to wind-up potentiation by ASICs and wind-up inhibition by ASICs. The dendritic calcium concentration and the membrane potential of the soma and dendrite are also shown. C and D are close-up views of A and B respectively, for a better comparison of the iCaAN current values. The higher ASIC conductance clearly correlates with higher calcium concentrations in the dendrite, as well as stronger activation of the hyperpolarizing KCa channels, which we postulate is the mechanism underlying windup inhibition by ASICs. Contrariwise, the increased calcium concentration in the dendrite does not cause much higher activation of the iCaAN channels (which would have the opposite effect). More fluctuations of the membrane potential in C lead to more current fluctuations, but currents fluctuate between roughly the same values the two conditions (i.e., C and D). This is because iCaAN channels are already close to saturation/full opening at the concentrations reached with the moderate ASIC conductance (gating variable m ~ = 0.78–0.92). (PDF) [file pcbi.1010993.s007.pdf]

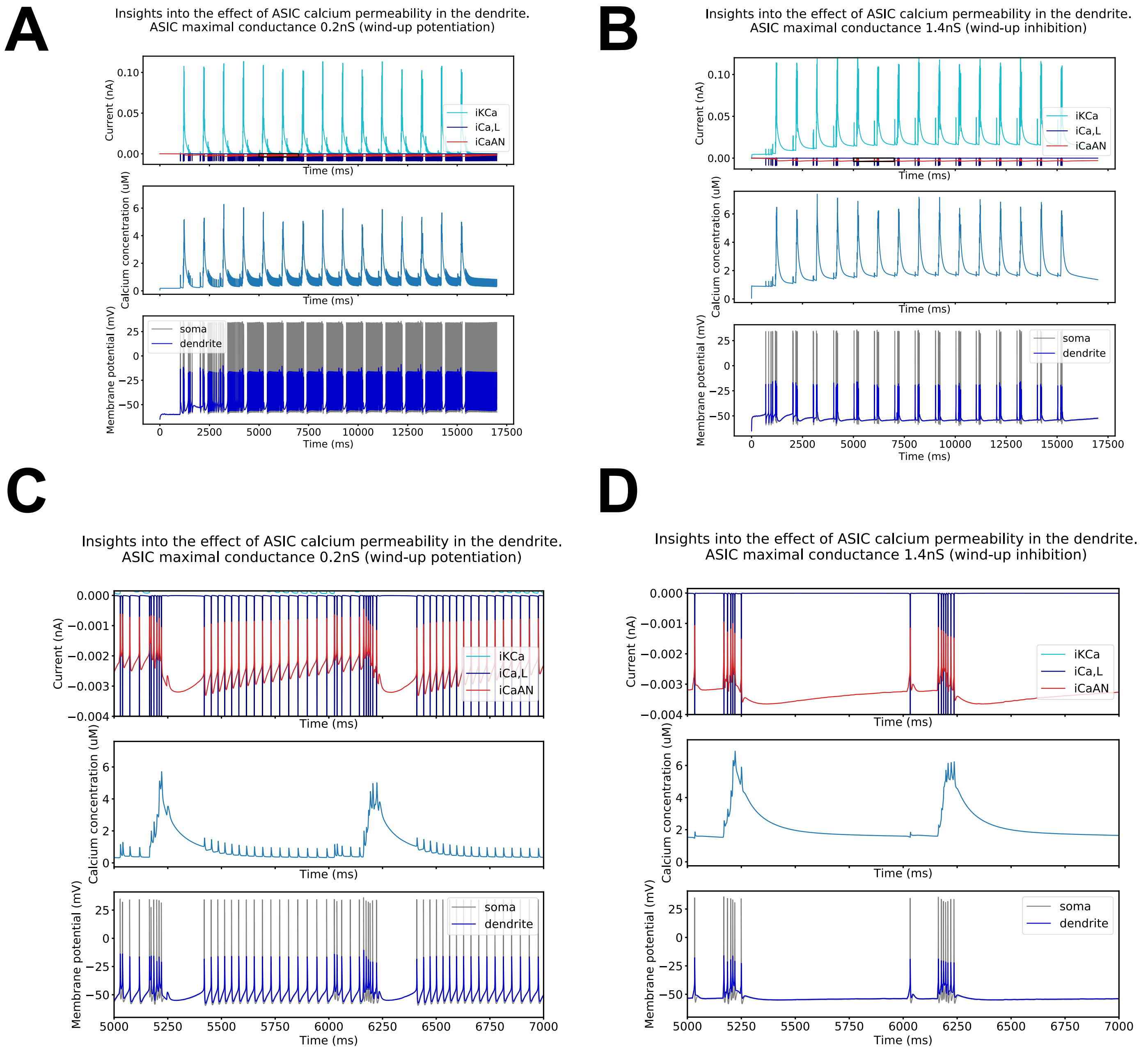

SUPPLEMANTARY FIGURE 7
