# Supplementary material for: Dual contribution of ASIC1a channels in the spinal processing of pain information by deep projection neurons revealed by computational modeling
Source: PLoS Comput Biol. 2023 Apr 17;19(4):e1010993. doi: 10.1371/journal.pcbi.1010993 (PMC10109503; doi:10.1371/journal.pcbi.1010993)
Supplement: Attachment — Submitted filename: Answer to Reviewers comments_Vfinale.pdf [file pcbi.1010993.s010.pdf]

Reviewer's Responses to Questions

Reviewer #1: This study investigates the contribution of ASICs to a neuronal plasticity mechanism in the spinal cord, termed windup, that leads to chronic pain. The authors show first with in vivo experiments that the windup is partially inhibited by application of each of two different ASIC inhibitors, Mambalgin-1 or PcTx1. Based on a patch-clamp characterization of neurons of lamina V of the dorsal horn, they propose that the ASICs in these cells are mostly heteromeric channels containing ASIC1a and ASIC2 subunits. The authors present then a computational model of WDR neurons, which can produce windup that depends on ASIC function. This model shows that at high ASIC current densities, windup can be inhibited. This is consistent with the effect of the ASIC activator MitTx that inhibits the development of a windup. Experimental inhibition of Ca-activated K channels prevents this effect of Mit-Tx. The authors suggest that ASICs contribute in a biphasic way to WDR neuron signaling, with potentiation at low, and inhibition at high ASIC activities.

Major points

1. Based on the pharmacology of the windup, the functional characterization of ASIC currents in dorsal horn neurons, and the kinetic models, the authors conclude that ASIC1a/ASIC2 heteromeric ASICs are responsible for the windup. The basis for this conclusion is however very weak. It contradicts the observation by the authors that windup is inhibited by PcTx1, which inhibits only ASIC1a homomeric channels at the experimental conditions used (see PMID 27277303). The functional characterization of ASIC currents in the neurons of lamina V shows inhibition by PcTx1, identifying them as mostly ASIC1a homomer-mediated currents. This contradicts the conclusion made.

⇨ Our initial conclusion of the involvement of ASIC1a/ASIC2 heteromeric channels was based on a combination of functional data in dorsal horn neurons compatible with such heteromeric channels and pharmacological evidence with PcTx1, mainly based on studies that have reported inhibitory effects of this toxin on both ASIC1a/ASIC2a and ASIC1a/ASIC2b channels, depending on the condition used (Joeres et al., Sci Rep 2016, doi: 10.1038/srep27647; Liu et al., Sci Rep 2018, doi: 10.1038/s41598-018-25386-9; Sherwood el al., J Neurosc 2011, doi: 10.1523/JNEUROSCI.1665-11.2011). However, we agree with the reviewer comments that the pharmacological evidence could be reinforced since (i) conditions used in most of these studies are different from the ones used in our study and, (ii) there is only one study reporting an inhibition in similar conditions and in a concentration range close to that observed for ASIC1a homomeric channels, with experiments performed in Xenopus oocytes. We thus performed additional experiments to test the effect of PcTx1 (30 nM and 300 nM) on HEK293 cells transfected with either ASIC1a and ASIC2b subunits, ASIC1a and ASIC2a subunits or ASIC1a subunit alone (new Fig. 3). We show that ASIC1a homomeric current (activated by a drop in pH from 7.4 to 6.0) was indeed the only one strongly inhibited by PcTx1, with no effects on ASIC1a/ASIC2b current (activated by a drop in pH from 8.0 to 6.0) and small potentiating effects on ASIC1a/ASIC2a current (activated by a drop in pH from 7.4 to 6.0, new Fig. 3C) as previously described. The discrepancy between our data on ASIC1a/ASIC2b and the study of Sherwood and colleagues (J Neurosc 2011, doi: 10.1523/JNEUROSCI.1665-11.2011) may be related to the fact they

performed their experiments in Xenopus oocytes while we performed our experiments in mammalian cells. Anyway, these new data now allow an experimental distinction between ASIC1a/ASIC2 heteromers and ASIC1a homomers and strongly support a role of ASIC1a homomers instead of ASIC1a/ASIC2 heteromers as initially proposed, in native ASIC1a-like currents recorded from WDR spinal neurons. The pharmacological experiments to investigate the effect of PcTx1 on homomers and heteromers are mentioned in the result section of our revised manuscript (page 7, lines 164-175) and the conclusion has been changed throughout the manuscript.

Regarding the experiments shown in Fig. 2, it is indicated that the conditioning pH was 7.4; in the methods it is said that the slices were kept at pH7.3. Was the measuring solution different from this storage solution? The authors should determine experimentally the pH dependence of the currents, since this parameter is very different between ASIC1a homotrimers and ASIC1a-2 heterotrimers and would allow an experimental distinction.

⇨ As indicated in the methods, spinal cord slices were stored in a chamber filled with normal ACSF bubbled with 95% O_2 and 5% CO_2, leading to a pH of 7.30-7.35. In the experiments shown in Fig. 2, the native ASIC current was induced by puffing recorded neurons with a pH6.6 solution from pH7.3. This point has been corrected in Fig. 2A and in its legend. Determining the pH dependence of native ASIC currents in spinal cord slices would have required the use of much more acidic pH (pH5.0 and below), which would have made it difficult to maintain neuron integrity in such a preparation. However, and as indicated above, new pharmacological data with the PcTx1 toxin allow now an experimental distinction between ASIC1a homotrimers and ASIC1a/ASIC2 heterotrimers in favor of a prevailing contribution of homomers.

2. In the model, a calcium permeability is attributed to heteromeric ASICs. The general view in the field is that only ASIC1a homotrimers are calcium permeable. Only one single study showed a calcium permeability of ASIC1a-ASIC2b channels. The authors should provide experimental proof of such a calcium permeability. In a heterologous system these would be straightforward experiments.

⇨ As indicated by the reviewer, only one study showed a calcium permeability of ASIC1a/ASIC2b channels. We therefore performed, as suggested, additional experiments in which we measured the pH6.0-induced intracellular calcium responses of HEK293 cells transfected with either ASIC1a and ASIC2b subunits, ASIC1a and ASIC2a subunits or ASIC1a subunit alone (new Supplementary Fig 2B, and see also new Fig. 3). These new data now demonstrate that, conversely to ASIC1a/ASIC2b heteromers, ASIC1a homomers and ASIC1a/ASIC2a heteromers are both able to induce a significant intracellular calcium rise in response to a pH6.0 extracellular acidification. This is now mentioned in our revised manuscript (page 13, lines 298-302), supporting a possible involvement of ASIC1a and/or ASIC1a/ASIC2a channels in the windup process. However, the new pharmacological data with the toxin PcTx1 introduced in the answer to point #1 support a prevalent contribution of ASIC1a homomers rather than heteromers.

3. More information about the model must be provided to allow the reader to better understand it and evaluate its quality. This should include description of the behavior of the two ASIC types in standard protocols, such as pH dependence of activation, inactivation, kinetics of inactivation, recovery from inactivation. The currents generated in the two ASIC models by the pH change shown in Fig. 3B should be shown.

⇨ The behavior of the two ASIC1a channel subtypes in response to standard protocols along with a short analysis is now described in Supplementary Figure 6A-D. The behavior of ASICs in response to the pH change of the simulation is now shown in Supplementary Figure 6E, and in the figure below (for the homomeric model). The x-axis is time in ms, the y-axis on the left (corresponding to the continuous line) shows the current in mA, and the y-axis on the right (corresponding to the dotted line) shows the conductance $g= \underline{g} *m*h$ in nS.

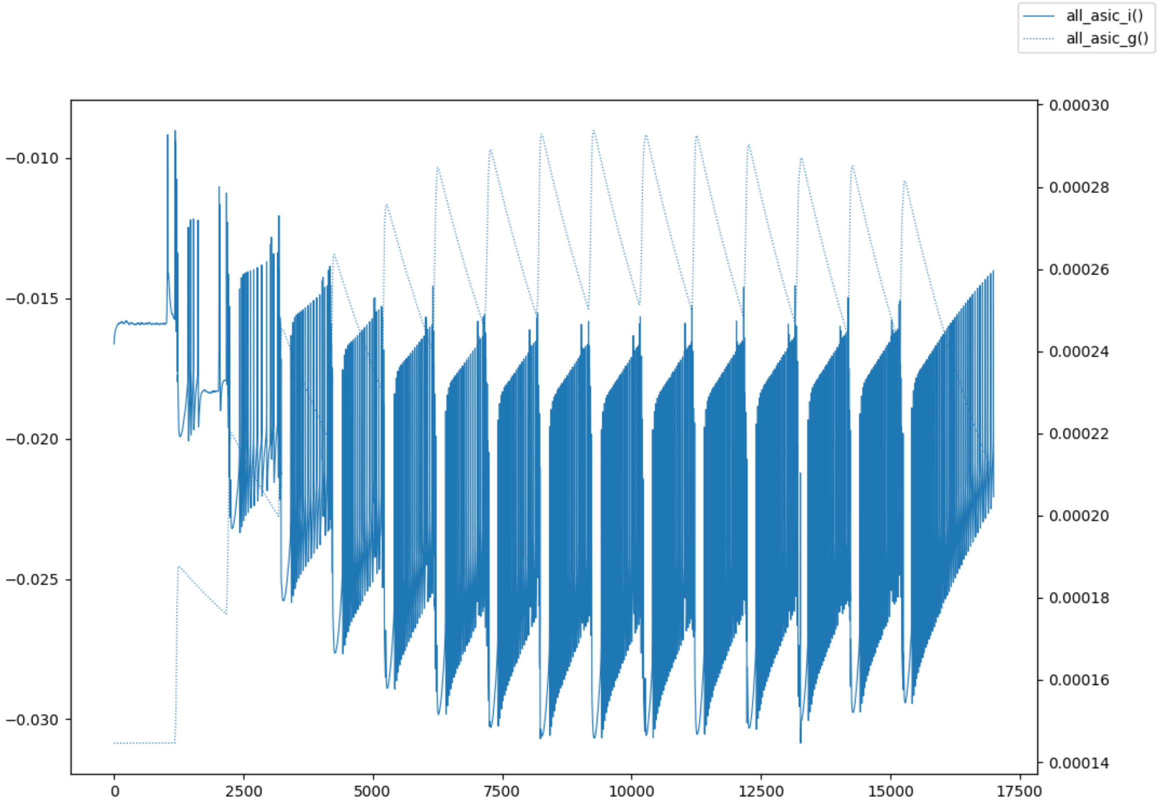

From their characterization of lamina V neurons in Fig. 2, the authors should estimate the current density of ASICs in these neurons; they should discuss how this value correlates with the different values that they used in Fig. 3C.

⇨ As requested by the reviewer, we estimated the global conductance of native ASIC1a currents recorded in WDR-like neurons (patch clamp experiments performed on spinal cord slices, Fig. 2). This global conductance was 1.9 nS, considering the mean pH6.6-evoked current amplitude recorded at -80 mV (-242 ± 37 pA) and a classical ASIC reversal potential estimated to +50 mV. This native ASIC1a conductance is well within the range of the conductances used in

our computational model (0 nS to 3 nS). This point is now mentioned in the revised manuscript (pages 9, lines 224-227). Note that as mentioned in the discussion, the exact values of ASIC-associated calcium influx, which is unknown, may alter the tipping point of the dual effect of ASICs: the less calcium they let in, the higher the conductance at which ASICs start activating KCa channels and reducing wind-up (in the high-conductance model as in the MiTx/full activation model.)

Clarify also in the legends whether the conductance values shown in Fig. 3 correspond to the maximal peak conductance that would be reached at the most acidic pH.

⇨ It is the maximal conductance $\underline{g}$ that would be theoretically reached if m=h=1; which never happens. This is now written in the figure legend.

In the description of the model in the methods, no properties of the A-delta fiber synapses are indicated.

⇨ The properties of A-delta synapses are the same as in the initial WDR model described by Aguiar and colleagues, and were kept unmodified in our study. A sentence has been added in the revised manuscript (second paragraph of "WDR neuron mathematical modeling including ASIC1a channel parameters and synaptic cleft acidification" in Material and Methods page 21, lines 495-497), to precise this point.

Spell out the name of the "NK1 receptor" and provide a reference.

⇨ NK1 is now spelled out in the text (page 4, line 73).

On p.19 in the methods, it is said that the ASIC1a homomeric model was unable to reproduce the experimental results. It would be good to indicate, due to which differences between ASIC1a homomers and heteromers the ASIC1a model could not produce a windup.

⇨ In our revised manuscript, we now provide different models of ASIC1a channels in WDR neuron, based on native ASIC1a parameters described in rat spinal neurons by Baron and colleagues (Baron et al., 2008, doi: 10.1523/JNEUROSCI.4975-07.2008) and on the ASIC1a model established by Allijevic and colleagues from non-native CHO transfected cells (Allijevic et al., 2020, doi: 10.3389/fncel.2020.00041). These ASIC1a models include the "type 2" (native ASIC1a heteromer) model provided in the initial manuscript, and a new "type 1" (native ASIC1a homomer) model. To our knowledge, "type 1" and "type 2" parameters are the only ones available describing native ASIC1a homomers and ASIC1a/ASIC2 heteromers in rat spinal cord neurons, respectively. We initially mentioned that the Alijevic model poorly reproduced our experimental data with no or little effect on windup. This is because no (q, τ) parameters could be found to observe results qualitatively similar to those shown in Figure 4C-F within a reasonable conductance range (small ASIC conductances hardly potentiate windup at all, and windup is not markedly reduced until maximal conductances increase to around 40nS). Indeed, the graphs of m and h as a function of pH are very sharp and have hardly any non-zero overlap, resulting in a very low activation of the ASIC1a Alijevic model. However, increasing the maximal conductance $\underline{g}$ by an order of magnitude to compensate for this showed that qualitatively similar results can be obtained with a conductance range from 0 to 45nS (data not shown), which was deemed

implausible. We therefore used only the two native models for our experiments in the revised manuscript. The comparison of the native homomeric and the heterologous homomeric models nonetheless suggests that the ASIC1a homomeric parameters may be slightly modified by their native environment (Baron et al., 2008) compared to heterologous systems (Allijevic et al., 2020). Anyway, these new computational data provide additional arguments for a participation of ASIC1a channels in WDR neurons to windup, with first a confirmation of experimental data and then similar predictions for the calcium–dependent windup decrease at high ASIC conductances (new Fig. 4). It is however difficult to distinguish from our computational models which subtype of ASIC1a channels are more likely to be expressed in WDR neurons (i.e., homomers or heteromers), but our new experimental data discussed in point #1 on the effect of PcTx1 on ASIC1a homomers and heteromers clearly support a major involvement of ASIC1a homomers. This is now stated in the text, page 8, lines 178-181.

The model of acidification of the synaptic cleft appears very hypothetical and based more on ASIC function than on information about pH changes. The amplitude of the pH change indicated in Fig. 3B appears to be much bigger than expected from most studies that investigated such pH changes. The authors should provide support from the literature for these predicted pH changes.

⇨ We agree that there is too little data available to build a very precise model of synaptic pH changes (many parameters of synaptic cleft acidification are unknown) but the pH changes used are within the range described in the literature (e.g., Chiacchiaretta et al. 2017 doi: 10.1242/jcs.198564). We now show that the model also works for smaller pH changes too (Fig. 4B) and does not rely heavily on the choice of pH parameters (Supplementary figures 4 and 9).

4. The most important findings of this study are experimental. I think that an experimental journal would be better adapted to the content of the manuscript.

⇨ We do not agree with the reviewer on this point since our data are not only based on experimental observations but also on the computational model that we described, which allowed to predict a negative unexpected participation of ASIC1a-type channels to windup process in a calcium-dependent manner, leading to the activation of KCa channels. This hypothesis would certainly not have been tested without the model, and we believe that this study would be of interest for readers of a biological / computational journal like PLoS Computational Biology.

Specific points

1. Contribution of calcium entry to the inhibition of windup at high ASIC activity. Could the authors determine with their models the time course of calcium entry in the different conditions used in Figs. 3C-D and 4C, and evaluate how the inhibition of the windup correlates with calcium entry? It should also be tested and shown in the model, whether for the induction of windup a calcium permeability of the ASICs is required.

⇨ The calcium permeability of ASICs is indeed not required to induce windup: first, windup is present even in the absence of ASICs; second, as seen in Figure 3D, ASICs potentiate the windup even without calcium permeability (and potentiate windup even at high conductances in the absence of calcium permeability).

⇨ We have added a supplementary figure 7 to discuss in more details how ASICs impact calcium concentrations and activate calcium-activated potassium (KCa) channels, which

depolarizes the neuron. Indeed, higher ASIC conductance results in a much higher calcium concentration in the dendrite, which correlates with higher KCa activation (and not really with increased calcium-activated non-specific current (iCaAN) activation, because these channels are already close to full activation for medium ASIC conductance) and inhibited windup.

2. p.5, upper part of the page, the description of the actions of PcTx1 and Mambalgin is somewhat misleading; PcTx1 inhibits heteromers only under very specific conditions that are different from the ones used in this study. This should be clarified.

⇨ This point is now clarified in the text page 5, lines 137-141 in light of the additional pharmacological experiments to test the effect of PcTx1 on ASIC1a homomers and heteromers in our experimental conditions, as also indicated in the answer to point #1 above.

3. Regarding the windup protocols, were different conditions (as e.g. control and different drugs) tested in the same animals? If yes, this should be mentioned in the methods, and it should be indicated how long the intervals between different tests were.

⇨ Yes, the different conditions were tested on the same animals. As mentioned in the methods, WDR neurons were stimulated every 10 min with brushings followed by a train of 16 supraliminar electrical repetitive stimulations. Controls and drugs were thus alternatively applied for 10 min between different stimulation protocols (brushings / electrical stimulations). Details have been added in the methods to clarify this point (page 17, lines 399-401).

4. p.9, 2nd paragraph, regarding Fig. 4B it is said that the effect of MitTx is fully reversible. However, the mean value after washout is probably close to 60% of the control value, thus this statement should be corrected.

⇨ We have added that inhibition by MitTx was "almost reversible upon washout (up to 50% of the control after 40 min of washout, Fig. 4B)". See page 10, lines 245-246.

5. p15, windup protocol, clarify to what the AUC measured in the windup protocols corresponds. Is it the AUC calculated from the graph of the number of APs as a function of the stimulus number?

⇨ Yes, the AUC for windup were calculated from the graphs of the number of APs as a function of the stimulus number (windup curves), and the baseline was set at the number of APs obtained at the first stimulation for each windup curve. These details have been added to the methods (page 17, lines 409-411).

6. Figure 1B, according to the text, the 16 stimulations in the windup protocol are applied at 1 Hz. In the figure however, the interval between the spikes does not correspond to 1s.

⇨ This is an error of the scale bar, which has been corrected in the revised manuscript.

7. For the toxin solutions, was there BSA used? Was BSA also included in the control conditions?

⇨ No, BSA was not used for in vivo experiments (see page 20, lines 474-478).

8. In Fig. 2A, 3 control applications of pH6.6 were recorded, plus 5 applications (applications 4-8) during the administration of the toxin. Control experiments should be shown in which applications 4-8 are recorded in the absence of any drug.

⇨ For the patch-clamp experiments performed on spinal cord slices, toxins were applied once a stable native pH6.6-evoked current was obtained, *i.e.* corresponding to at least three currents with similar peak amplitudes, as indicated in figure 2A. Moreover, we show in figure 2C that the native pH6.6-evoked current does not display any run down (tachyphylaxis) of its amplitude over 4 consecutive stimulations, indicating that the inhibitions observed can be attributed to the toxins.

9. Could you also describe the experimental conditions for ASIC1a-expressing HEK cells? Indicate also whether the recombinant ASIC1a was from rat.

⇨ Experiments on HEK293 cells were performed using the rat clones of ASIC1a, ASIC2a and ASIC2b. This is now stated in the methods of the revised manuscript with a specific paragraph entitled “Patch-clamp and Calcium experiments on HEK293 transfected cells” (page 18-19, lines 433-468).

10. Indicate in the figure legends the timing of drug applications; indicate whether they were applied only during the windup protocols, or if there was a pre-application, how long this was.

⇨ The timing of drug applications is now indicated in each figure legend.

11. Supplementary Figure 1, indicate exactly what is meant with "control 1 and 2". Should the PcTx1 concentration in the legend rather be 30 nM?

⇨ Control 1 and 2 represent two brushing experiments that were performed consecutively at 10 min intervals before application of mambalgin-1 or PcTx1 for 10 min. This is now stated in the legend of supplementary figure 1B.

I am somewhat lost in panel C. I understand that stimulations of the windup protocol are applied once per second. However, the traces shown in panel C are much longer than 1s.

⇨ There was an error with the scale bar in supplementary Fig. 1C. As also requested by reviewer 2, the traces shown have been changed with an appropriate time scale bar.

12. The supplementary figure is very difficult to understand. There are 3 conditions mentioned in the legend, however only 2 of them have an attributed color. In the graphs, there are colored lines and colored surfaces. There is a purple surface whose meaning is not defined.

⇨ It is difficult to answer this specific point since the figure number is not mentioned. However, we suppose this comment is about grid search graphs (Supplementary Fig. 4). The colors have been changed, but more importantly each condition has an attributed color and the meaning of the overlapping colors has been clarified by illustrative overlapping circles (the non-attributed colors result from the overlapping of two or more attributed color zones). The lines only outline the boundary of each color zone for more clarity.

<u>Reviewer #2</u>: The manuscript by Chafai et al provides functional evidence for a role of the ASIC1a subunit in the process of “wind-up” in WDR neurons in lamina V of the spinal dorsal horn in rats. Wind-up measured as a progressive increase in evoked activity in WDR neurons was induced with electrical stimulation at C-fibre intensity. Wind-up was reduced by ASIC inhibition with PcTx-1 and Mambalgin-1. Whole cell recordings from large diameter neurons in lamina V showed inward currents in response to pH challenge to 6.6 that were blocked by both PcTx-1 and Mambalgin-1. Numerical simulations indicate that a progressive fall in pH leads to an increase in an ASIC conductance that can produce firing patterns similar to those observed with wind-up. Interestingly, application of MitTx, an activator of ASIC1a, also reduced wind-up. To reconcile this apparent paradox, a permeability of ASIC1a to calcium was postulated to lead to activation of calcium-activated potassium channels and thus a reduction in wind-up. A role for calcium activated potassium channels was confirmed by experiment and simulation.

This is an interesting manuscript that provides a functional characterization of putative ASIC currents in WDR neurons. Pharmacological blockade of ASIC reduced wind-up and this experimental result was consistent with results from numerical simulations using an ASIC conductance. In contrast, pharmacological activation of ASIC also reduced wind-up and to explain this, the authors postulate calcium entry via ASIC1a heteromers and ensuing hyperpolarization via IK and BK. This second dataset requires additional work to substantiate the involvement of calcium as outlined below.
The Figures present the data clearly and the manuscript is generally well written.

Major points
1. There is a striking dichotomy in that both blockade and activation of ASIC reduce wind-up. The authors recognise this point and take up an explanation in the second paragraph of the discussion. However, the explanation provided here is ambiguous and conflates several ideas (outlined below). From my perspective to clarify the role of calcium additional experimental evidence is required to support the idea of calcium entry via ASIC in WDR neurons.To re-iterate, toxin blockade of ASIC reduced wind-up. This implies that ASIC contributes to the wind-up process in the following manner. An ASIC conductance is low at rest and then during wind-up, as synaptic pH falls, ASIC is progressively recruited to enhance the wind-up process. The toxin blockers PcTx-1 and Mambalgin-1 both prevent this. However, toxin activation of ASIC with MitTx also reduced wind-up. Assuming the same starting conditions, with a low ASIC conductance at rest, channel opening with MitTx should initially enhance wind-up. It does not. Rather, the clear result from the concentration response profile in Figure 4A&B is that MitTx either did not affect or reduced wind-up. This is an intriguing result and since sits at the crux of the manuscript. To explain reduced wind-up with ASIC activation by MitTx, the authors postulate that MitTx opens an ASIC conductance permeable to calcium. In the same sentence however, the idea of maximal channel activation is brought up by stating that “the consequence of their over-activation (MitTx effect) was unexpected and associated to the calcium conductance of ASIC1a-containing channels. (pp.11, last 3 lines). Two concepts are conflated here, specifically (1) maximal channel opening and (2) calcium permeability of ASIC. Taking these two ideas in turn. Firstly, maximal activation leads to calcium entry. The problem here is that the concentration-response profile for MitTx shown in Figure 4 demonstrates that MitTx never potentiates wind-up. This would imply that

MitTx, even at low concentrations, maximally opens ASIC and this seems to be unlikely. To be specific, how does the ASIC at rest get to the maximally activated and thus calcium permeable state without showing some evidence of increasing the Na influx assigned to it during the process of wind-up.?? In addition, why does the calcium permeability not manifest during electrically evoked wind-up, i.e. without toxins under normal circumstances? The second idea of calcium permeability is not clearly formulated in the text. My interpretation is that there are multiple sub-types of ASIC channels in WDR neurons and this is likely. In which case, MitTx activates an ASIC channel population comprising splice variants such as ASIC1a/ASIC2a that have a predominant Ca-conductance. In contrast, the ASIC channels recruited by electrical stimuli (and blocked by PicTx and Mambalgin-1) represents an ASIC population with low Ca-permeability – is this the case? The discussion does not adequately clarify this. Irrespective of the interpretation I would like to see experimental evidence demonstrating an increase in calcium in WDR neurons, at least in response to MitTx and better yet a smaller or absence of calcium increase during wind-up to electrical stimulation. This could be done using a calcium reporter. One might expect that during electrical wind-up the calcium increase is modest while in response to MitTx a prominent calcium increase ensues. Increases in calcium via calcium channel activators or perhaps thapsigargin mediated store release might also be valuable in this context.

⇨ It would be great to see calcium variations in WDR neurons during windup but, in addition to be quite challenging experiments, it would be difficult to discriminate the relative contribution of ASIC channels to the whole increase in calcium associated with neuronal activation. Calcium permeability of ASICs is however a key point in our hypothesis of an involvement of Ca^{2+} through these channels, and we have performed additional experiments to assess the calcium permeability of ASIC1a/ASIC2b channels, because this was described in only one single study and may be questionable. We measured the pH6.0-induced intracellular calcium responses of HEK293 cells transfected with either ASIC1a and ASIC2b subunits, ASIC1a and ASIC2a subunits or ASIC1a subunit alone (new Supplementary Fig 2B, and see also new Fig. 3). These new data now demonstrate that, conversely to ASIC1a/ASIC2b heteromers, ASIC1a homomers and ASIC1a/ASIC2a are able to induce a significant intracellular calcium rise in response to pH6.0 extracellular acidification. This is now mentioned in our revised manuscript (pages 13, lines 301-305), further supporting, together with new pharmacological evidence showing no inhibition of ASIC1a/ASIC2 currents by PcTx1 in our conditions (contrary to a previous study and mentioned in the result section of our revised manuscript, page 8, lines 178-181), the involvement in the windup process of ASIC1a homomeric channels instead of ASIC1a/ASIC2 heteromers as initially proposed.

⇨ About the *in vivo* effect of MitTx, the reviewer suggests that one would have expected to see (i) positive effects on windup for low toxin concentrations and (ii) negative effects on windup for higher toxin concentrations, and that these effects would have been associated with low and high activations of ASICs by MitTx, respectively. This would make sense in light of the calcium-dependent windup decrease predicted by the model for high ASIC conductances. However, the mathematical model is based on the physiological activation of ASIC1a channels by their natural ligand (protons), i.e., transient currents generated by activation and inactivation upon acidification, whereas the MitTx is a constitutive activator of ASIC with almost no inactivation (ASIC1a stays open as long as the MitTx is present; see Bolhen et al., Nature 2011 doi:10.1038/nature10607). So even if low concentrations of MitTx do not maximally activate ASIC1a, but rather have a

progressive and dose dependent activating effect, the constitutive non-physiological nature of this activation may probably be an explanation to the windup decrease observed. Indeed, the non-inactivating ASIC current generated by MitTx, even at low concentrations, is associated with a constitutive Na^+ and Ca^{2+} entry into neurons that will have drastic impact on intracellular calcium activity susceptible to significantly affect the windup. This point is now indicated in the discussion page 13, lines 293-301.

Why does the calcium permeability not manifest during electrically evoked wind-up, i.e. without toxins under normal circumstances

⇨ The calcium permeability is there but because the conductance is low, the calcium influx is nonetheless too small to significantly affect the windup. This is well supported by our simulation data, which show that windup increases upon removing the calcium permeability of ASICs (Fig. 4D and 4F).

2. Although it is stated in the text on pp.9 (second to last sentence) that apamin and iberotoxin in combination do not affect wind-up - it would be important to show this data as part of a Figure.

⇨ A new supplementary figure, which has been extracted from Fig. 5D, is now specifically showing the effects of apamin and iberiotoxin (new supp. Fig. 5).

3. Drugs were applied to the entire spinal cord and thus any number of neurons, including axons from afferents are equally likely to be affected. The question then is, to what extent is the reduction in wind-up with both PcTx-1 and Mambalgin-1 not due to blockade of WDR neurons directly but rather due, at least in part, to blockade of primary afferent signals? The authors point out that both toxins reduced the response to the first stimulus and acknowledge that this represents a reduction in primary afferent input. How can one dissociate at which site the reduction in wind-up is taking place? and can this be done in a quantitative manner, eg. 40% reduction in primary afferent input and 20% from WDR?? This seems to be a major shortcoming of the manuscript.

⇨ We fully agree with the reviewer that drugs used *in vivo* were applied to the spinal cord at the laminectomy level, and that different types of neurons can be affected beside WDR. However, the complementary computational modeling approach brings a proof of concept that ASIC1a channels in WDR neurons are potentially able to participate to windup. Anyway, this does not exclude that the experimentally observed drug effects may also be associated with other spinal neurons, including axon afferents. This point is now stated in the discussion of the revised manuscript (page 12, lines 274-279). The toxin-evoked decrease of the response to the first stimulus can be supported by effects at different levels including axons from afferents, and therefore does not necessarily represent only a reduction in primary afferent input, precluding any quantitative assessment of the wind-up inhibition nature.

4. The summary figures show paired data with values before and after drug application (eg. 1C&Finsets; 1D,E,G&H; 2D; 4E; Supp1AB&D). To better visualise the paired nature of these datasets, it would be helpful to join pairs of dots with a line. Showing independent clouds of data points is misleading for paired data.

⇨ Figure 1 has been modified so that paired data are now represented as suggested by the reviewer.

Minor points:

1. Is the reference to Liu et al (2018) cited in the discussion pp.11 (beginning on last line) “This latter point strongly argues for an expression in WDR neurons of heteromeric ASIC1a/ASIC2b channels, which have a significant Ca2+ permeability [38].” Correct because Liu et al (2018) does not show that ASIC1a/ASIC2b channels have an elevated Ca-permeability.

⇨ This has been removed as the discussion has been significantly modified (pages 12-14) to discuss the MitTx effect and also ASIC Ca^{2+} permeability in light of our new experiments on homomeric and heteromeric ASIC1a channels.

2. The methods state on pp. 15 (Windup protocol and analysis paragraph, last sentence) ”Area under curves (AUC) for windup curves were determined using Prism software.” and I assume this means simply the integral of AP counts over time. Is this correct? and could this procedure be explained in more detail?

⇨ Yes AUC means the integral AP counts over the number of stimulation. Details on how AUC were calculated have been added in the methods (page 17, lines 409-411).

3. The species should be mentioned earlier, either in the title or the abstract.

⇨ The species from which experimental data were obtained is now mentioned (i.e., rat) in the abstract (page 2, line 26).

4. Supplementary Figure 1, panel C shows prominent 50Hz noise in the signal – would it be possible to present a cleaner recording.

⇨ The recordings shown in the panel C has been changed.

Reviewer #3: Windup is an important pain facilitation process in the spinal dorsal horn, characterized by a progressive, frequency-dependent increase in the excitability of WDR nociceptive neurons to repetitive stimulation of primary afferent nociceptive C-fibers. Windup plays an important role in spinal neuronal plasticity and is of unquestionable interest to understand pain processing in the dorsal horn. As the authors point out, the exact mechanism(s) behind windup is(are) still not fully understood.

Throughout the years (windup was originally reported ~half a century ago), different groups have been revealing different contributors for this facilitation process. Not so many groups have taken an integrative approach providing a holistic analysis where the effective role of different contributors are weighted. It should be noted that, as 1 Hz is the typical stimulation frequency in windup protocols, any mechanism with a "recovery" time constant/scale larger than 1 sec will potentially contribute to signal integration and an observable effect on windup profiles.

In this work the authors point to yet another contributor for action potentials windup in WDR neurons: acid sensing ion channels.

This is a clear and well-written manuscript combining in silico, ex vivo and in vivo experiments to verify and assess the contribution of ASIC currents is action potentials windup in WDR neurons.

Questions

1) The choice of the dynamics for the synaptic cleft H+ concentration is critical in shaping the windup profile. How is the time scale of the acidification "windup" (Fig. 3B) related to the time scale of the windup in the number of APs? In other words, is the APs windup profile (time constant) the same as the pH profile (time constant)?

⇨ We would like to point out that there is windup without ASICs and therefore without pH variations. We can only be qualitative with this model because many parameters of synaptic cleft acidification are unknown. Indeed the time scale of the acidification shown in Fig. 3B (now Fig. 4B) happens to be similar to that of windup, but we nonetheless show in the supplementary figure 9 that the qualitative effect of ASICs on windup (potentiation then inhibition as maximal conductance increases) does not depend on the time scale of the acidification, as similar results can be achieved with an acidification at both long and short time scales: acidification is crucial to activate ASICs, but its precise dynamics does not appear to be so.

2) No need for a sensitivity analysis on the new ASIC model + ProstProtCleftDyn model, but some insight would be relevant/useful on how robust the conclusions are regarding changes in the parameters of these new models.

⇨ This is the subject of supplementary figure 4, which answers the question for the case of the synaptic cleft pH model and some parameters of ASICs. Please note that the figure sums up several key factors for the windup profile for different ASIC models.

3) Regarding this point "This study demonstrates a possible dual contribution to windup of calcium permeable ASIC1a/ASIC2 channels in deep laminae projecting neurons, promoting it upon moderate channel activity, but ultimately leading to calcium-dependent windup inhibition associated to potassium channels when activity increases". As far as I understood, your model

includes both Ca-dependent potassium currents and non-specific current dependent on intracellular Ca concentration (iCaAN). According to Fig 3D, iCaAN in your model does not seem to be needed to provide the temporal integration supporting windup. If iCaAN is not needed, I would state that.

⇨ Our model is based on a WDR model already published by Aguiar and colleagues (Aguiar et al., 2012, doi: 10.1152/jn.00834.2009) in which we added different ASIC1a parameters and a model of synaptic cleft acidification. We only slightly modified the initial Aguiar model by removing an interneuron to better fit our experimental windup (see supplementary fig. 3), but we tried to keep all the other parameters as described in the original paper. As iCaAN is mentioned as crucial in the Aguiar model (Aguiar et al., 2010, doi: 10.1152/jn.00834.2009), we did not try to modify or remove it. In our figure 3D (now 4D), iCaAN still participates to wind-up: the calcium permeability of ASICs has been abolished, but not other calcium-permeable channels and receptors. However, as shown below in the answer to point #5 regarding how ASICs compare with other currents playing a role in windup, ASIC currents appear to have a much bigger amplitude than iCaAN in the model. This is now stated in the discussion page 14, lines 319-324.

4) I would argue that “total number of AP” nor “area under curves (AUC)” are the best metrics to characterize (and compare) windup curves. The ratio between the (asymptotic) maximum number of APs and the number of APs in response to the first stimulation seems to be a better metric. It is more robust for comparisons, especially in curve comparisons were the number of APs in the first response is different.

⇨ The ratio between “maximum number of APs and the number of APs in response to the first stimulation” proposed by the reviewer could be a good way to represent windup but it would be very difficult with our data since the number of APs at first stimulation sometimes fall to zero following application of toxins (not possible to divide by 0). However, the AUC that we used have been calculated using Prism software from windup curves (number of APs as a function of the number of stimulation), and the baseline was set at the number of APs in the first response for each windup curve. Moreover, we used three different and complementary ways to represent windup, including the classical windup curves (number of APs as a function of the number of stimulation), the total number of APs evoked during the 16 stimulations of a windup protocol, and the AUC.

5) For the sake of advancing the understanding on the facilitation process of windup, it would be important to have in the Discussion more information on how ASICs compares with the other currents playing a role in windup (e.g. NMDA receptors, NK1 receptors, and L-type calcium channels). Are ASICs necessary and/or sufficient to produce APs windup? This should be discussed taking into account a potential therapeutic strategy based on ASICs inhibition.

⇨ Our data clearly show that ASICs, contrary to NMDA receptors for instance, are not mandatory to produce APs windup since their pharmacological inhibition by the toxins PcTx1 or mambalgin-1 decreases, but not abolishes, windup. ASIC1a thus seems to have a rather modulatory effect. The model indeed allows to make hypotheses on how ASICs compare with other currents playing a role in windup. The figures below (which are our new Supplementary fig. 8) show the currents in the dendrite during the simulation. The first figure shows the ASIC current in blue and the sum of all other trans-membrane currents in the dendrite over the whole simulation;

for a better understanding, the second and third figures are close-ups with, in addition, each of these currents shown separately.The AMPA and NMDA currents clearly dominate, as expected, the input to the dendrite.
The ASIC current, shown in blue, is a sustained and increasing current. Its dynamic is rather similar to that of the NK1 receptor associated currents although the currents are bigger; this may explain that at low to moderate conductances, ASICs participate to windup as NK1s do (as long as the associated calcium influx is not too big).
The ASIC currents also have a much bigger amplitude than the calcium-activated non-specific currents iCaAN.
The dynamics of the L-type calcium channels, only activating briefly at spike times, is quite different from that of ASICs, which might also explain why the calcium influx from ASICs may inhibit windup although L-type calcium channels participate to windup generation according to Aguiar *et al.* (Aguiar et al., 2010, doi: 10.1152/jn.00834.2009).
This is now stated in the discussion pages 13-14, lines 312-324.

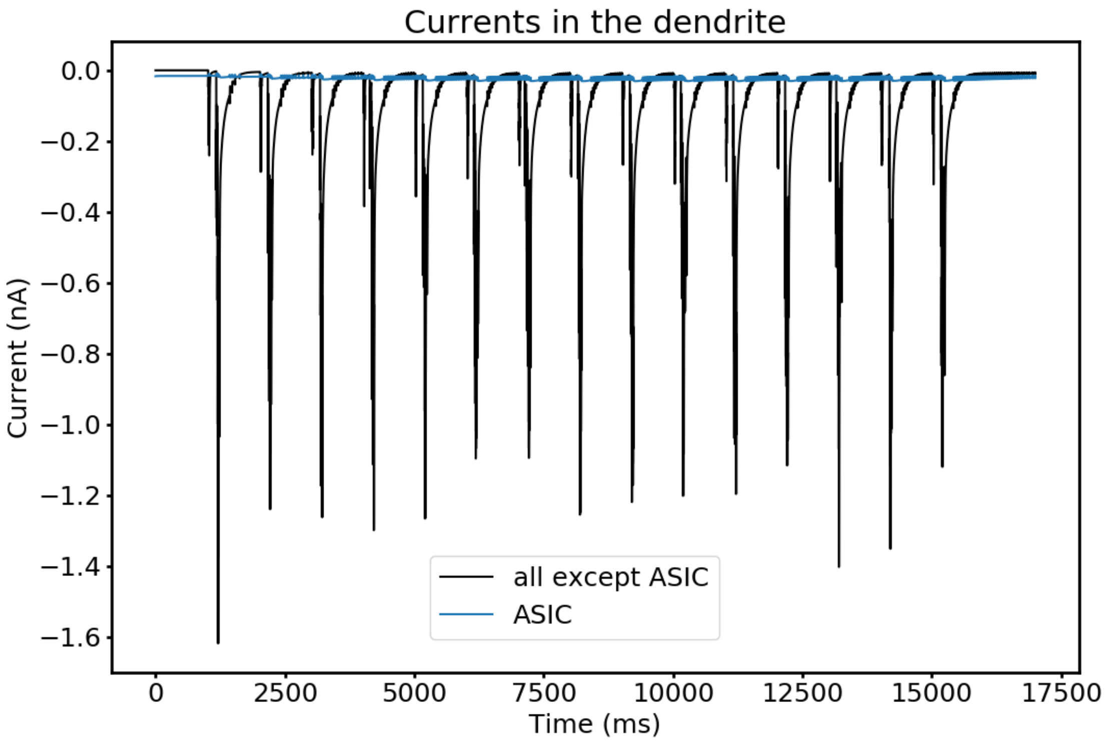

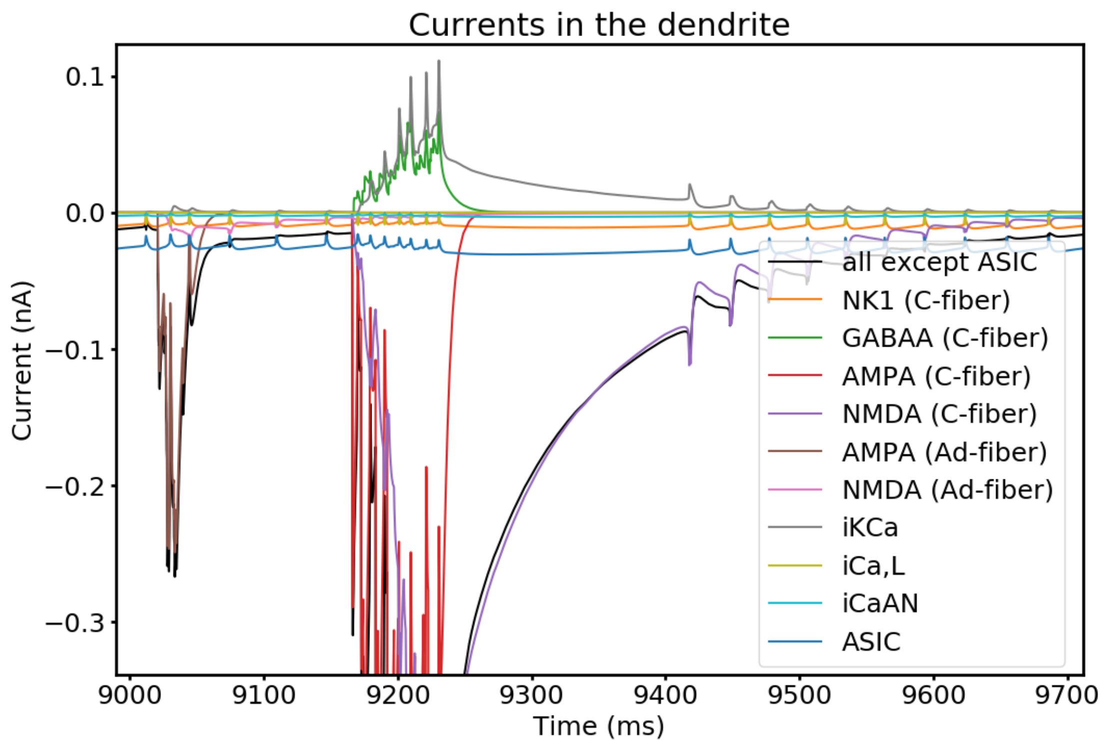

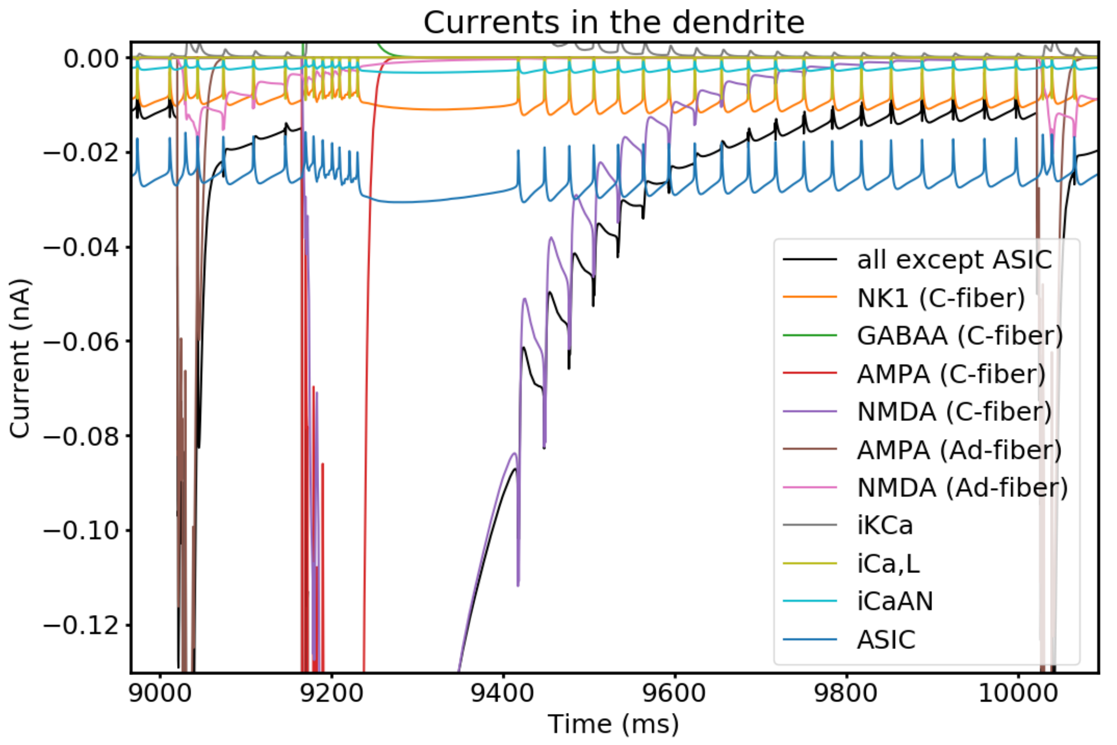

Minor issues:

- Inconsistent use of “Fig” and “Figure”
    - ⇨ This has been homogenized.

- “was decrease by 20% in the presence of mambalgin-1” -> “decreased”
    - ⇨ This has been corrected.
- When running the NEURON simulation (using mosinit.hoc file), the title of the window should be corrected (it retains the name of a previous publication).
    - ⇨ Done
- I would suggest that mosinit.hoc would already produce a figure with the .dat file. But this is just a suggestion thinking on a colleague that, for a quick test, may want to run the simulation without a NEURON+Python installation
    - ⇨ Thank you for the suggestion, we will consider it.
