# Supplementary material for: Dual contribution of ASIC1a channels in the spinal processing of pain information by deep projection neurons revealed by computational modeling
Source: PLoS Comput Biol. 2023 Apr 17;19(4):e1010993. doi: 10.1371/journal.pcbi.1010993 (PMC10109503; doi:10.1371/journal.pcbi.1010993)
Supplement: Attachment — Submitted filename: Answer to reviewers_rev2.pdf [file pcbi.1010993.s011.pdf]

Answer to reviewer's comments

1. In Fig. S6E, the relative conductance m*h under the synaptic cleft acidification pattern is displayed. These conductances seem low and extremely low for the homomeric and heteromeric models, respectively. To allow the reader to have a point of comparison, the authors need to indicate (in the text or the legend) the m*h value that is reached at the peak following a typical ASIC1a-activating pH change, such as from pH7.4 to pH6.0 for the ASIC1a homotrimer and for the ASIC1a-containing heteromer model.
As suggested, we added a panel in supplementary figure 6 (Fig.S6F) showing the m.h values reached following a pH change from 7.4 to 6.6 for both homomeric and heteromeric ASIC1a channels.

2. Fig. S7 is very useful for the understanding of the model. Based on this figure it is concluded that the higher ASIC conductance in B/D does not change much the ICaAN. The upper panel of C is however almost completely red and green, very different from the corresponding panel in D. Does the high color content in C indicate that ICaAN and ICa,L are fluctuating at a high frequency between different current amplitudes, while in condition D, ICaAN reaches soon after each spike a maximal inward current amplitude, from which it decreases slowly until the next spike?
The high color content in C indicates rapid current fluctuations induced by rapid potential fluctuations (high spike frequency). Because the spike frequency is lower in D, with calm time zones between the stimulations, there are no such fluctuations of the currents. But the current peaks reach roughly the same amplitudes in both conditions, that is what we meant by saying that ICaAN is not changed much.

And ICa,L would be active only during the spike?
Yes, ICa,L is only active during spikes, in both conditions.

The quality/resolution of this figures is not good enough to see any details. The authors need to better describe and interpret this model behavior in the text of the manuscript.
We now provide better quality/resolution graphs and we added some explanations in the legend of Fig. S7: "Contrariwise, the increased calcium concentration in dendrite does not cause much higher activation of the iCaAN channels (which would have the opposite effect). More fluctuations of the membrane potential in C lead to more current fluctuations, but currents fluctuate between roughly the same values in the two conditions (i.e., C and D)."

The plots of Ca concentrations are the same in A and C, and B and D. It is not necessary to show them twice. However, it is important to show the membrane potential under these protocols, since the windup is determined as a change in the number of action potentials after each electrical stimulation. This could be done, for the two conditions, in either Fig. S7 or S8, over the whole time of a windup protocol.
This is done in Fig. S7, thank you for the remark.

3. The resolution of most supplementary figures is very low. This may be due to the merging of several files for review. The authors should make sure that the uploaded supplementary data files are of sufficient quality.
We have exported the supplementary figures in high quality pdf, which allows for zooming on specific parts of the graphs.

4. p10, line 245, "Inhibition of windup by MitTx was almost reversible....", more correct would be "Inhibition of windup by MitTx was partially reversible....".
Thank you, we corrected it accordingly.

5. Fig. S9, the unit in the x-axis label should be s, not ms. In this figure, the resulting ASIC currents induced by the two pH patterns of A and D should be shown.
Thank you for spotting the mistake. We also implemented the figure with two new panels (D and H) to answer your suggestion.

6. Overall it seems that under the conditions of the windup protocol, the ASIC currents are very low but still contribute importantly to the windup. This aspect should be discussed. Could there be any parallels between this function and the role of ASIC activity in LTP, where ASIC currents are also very small?
This point is illustrated in Fig. S8. A sustained current, even of low amplitude, is a powerful depolarizing force compared to short transient currents. ASICs, in our simulation, have dynamics similar to NK1 receptors, which are known to potentiate wind-up, with even stronger currents.
We agree with the reviewer that, although different, windup (short-term sensitization) and LTP (long-term potentiation) are associated with synaptic plasticity and share some similarities. ASIC1a has, indeed, already been involved in LTP in different brain areas including amygdala (Du et al., PNAS 2014, doi: 10.1073/pnas.1407018111 ; Chiang et al., Sci. Rep. 2015 ; doi: 10.1038/srep10143) and cingulate cortex (Gobetto et al., Neurosc. 2021, doi: 10.1016/j.neuroscience.2021.01.022). The involvement of ASIC1a in LTP could thus be related to the current generated by repetitive stimulations, as suggested here for windup, although the stimulation frequency that induces windup and LTP are different, *i.e.*, low frequency for windup and high frequency for LTP. This is now discussed in the text of the manuscript page 14: "The small but sustained ASIC current induced by repetitive stimulation appears to be important for the role of ASIC1a in windup, as it may also be the case for its role in LTP that have been reported in different brain areas (Du et al., PNAS 2014 ; Chiang et al., Sci. Rep. 2015 ; Gobetto et al., Neurosc. 2021), although the two processes are different".
